# Impact of Chromium Picolinate on Leydig Cell Steroidogenesis and Antioxidant Balance Using an In Vitro Insulin Resistance Model

**DOI:** 10.3390/antiox13010040

**Published:** 2023-12-23

**Authors:** Rúben Moreira, Ana D. Martins, Rita Ferreira, Marco G. Alves, Maria de Lourdes Pereira, Pedro F. Oliveira

**Affiliations:** 1 Department of Chemistry, University of Aveiro, 3810-193 Aveiro, Portugal; rubenjesusmoreira@ua.pt (R.M.); anacdmartins@gmail.com (A.D.M.); ritaferreira@ua.pt (R.F.); 2 LAQV-REQUIMTE, University of Aveiro, 3810-193 Aveiro, Portugal; 3 iBiMED-Institute of Biomedicine, Department of Medical Sciences, University of Aveiro, 3810-193 Aveiro, Portugal; alvesmarc@gmail.com; 4 CICECO-Aveiro Institute of Materials, University of Aveiro, 3810-193 Aveiro, Portugal; mlourdespereira@ua.pt; 5 Department of Medical Sciences, University of Aveiro, 3810-193 Aveiro, Portugal

**Keywords:** chromium picolinate, trivalent chromium, Leydig cells, steroidogenesis, reactive oxygen species, antioxidants

## Abstract

Leydig cells (LCs) play a pivotal role in male fertility, producing testosterone. Chromium (III) picolinate (CrPic_3_), a contentious supplement with antidiabetic and antioxidant properties, raises concerns regarding male fertility. Using a rodent LC line, we investigated the cytotoxicity of increasing CrPic_3_ doses. An insulin resistance (IR) model was established using palmitate (PA), and LCs were further exposed to CrPic_3_ to assess its antioxidant/antidiabetic activities. An exometabolome analysis was performed using ^1^H-NMR. Mitochondrial function and oxidative stress were evaluated via immunoblot. Steroidogenesis was assessed by quantifying androstenedione through ELISA. Our results uncover the toxic effects of CrPic_3_ on LCs even at low doses under IR conditions. Furthermore, even under these IR conditions, CrPic_3_ fails to enhance glucose consumption but restores the expression of mitochondrial complexes CII and CIII, alleviating oxidative stress in LCs. While baseline androgen production remained unaffected, CrPic_3_ promoted androstenedione production in LCs in the presence of PA, suggesting that it promotes cholesterol conversion into androgenic intermediates in this context. This study highlights the need for caution with CrPic_3_ even at lower doses. It provides valuable insights into the intricate factors influencing LCs metabolism and antioxidant defenses, shedding light on potential benefits and risks of CrPic_3_, particularly in IR conditions.

## 1. Introduction

Infertility is the inability to achieve pregnancy after 12 months or more of regular unprotected sexual intercourse [1]. This condition may be of male and/or female origin [2], with an estimated 30 million men suffering from infertilityworldwide [3]. Continuous exposure to endocrine-disrupting chemicals (EDCs) has an impact on both human health and the environment. It is a key factor for the reported high infertility rates, since they interfere with the endocrine signaling of the body [4,5]. Among the hormonal dysfunctions induced by EDC exposure, testosterone production, crucial for male reproductive system functionality and spermatogenesis initiation, is particularly sensitive [6]. Leydig cells (LCs), one of the primary somatic cells in the testes, contribute approximately 95% of circulating testosterone in adult males [7,8].

Heavy metals, such as chromium (Cr), are considered EDCs with known toxicological risk to sexual health and male fertility, by affecting semen quality parameters and the secretory function of accessory sexual glands [9]. Cr can be found in nature in two forms: hexavalent Cr (Cr(VI)) and trivalent Cr (Cr(III)) [10]. Cr(VI) has been proven to be a risk for human health, being associated with nephrotoxicity [11], hepatotoxicity [12], cancer [13], and structural and functional damage in the testes [14,15,16]. Because of this, Cr(VI) has been the focus of the scientific community for longer than Cr(III). Nevertheless, some studies on Cr(III) are raising concerns regarding its safety [17] and even challenging its classification as an essential element [18]. Amongst the various Cr compounds, chromium picolinate [tris(picolinate)chromium(III)] (CrPic_3_) has become a very popular supplement [19] to reduce weight or manage blood glucose levels [20]. Other uses described for this supplement include the treatment of depression [21,22], protection against heat stress [23], stimulation of ovulation in women with polycystic ovary syndrome, improvement of lipid profile [24], and antioxidant effects in cellular systems, such as enhancing the activity of antioxidant enzymes and decreasing damage caused by oxidative stress, particularly lipid peroxidation and protein carbonylation [25,26,27,28]. When it comes to the impact of CrPic_3_ on male fertility, the research landscape reveals conflicting findings. Some studies suggest that Cr(III) does not impact testosterone production; others suggest that it can decrease testosterone production and affect LC integrity [29,30]; others show that it increases testosterone production [31,32]; and others suggest that the damages observed in LCs can be a technical artifact [33]. Still, available studies imply that LCs are a target of CrPic_3_, with their action in these cells still far from being fully understood (refer to [34] for details). Hence, we conducted a study to further clarify the effects of CrPic_3_ on male fertility, aiming specifically to discover its impact on LC physiology and testicular steroidogenesis in an in vitro model of insulin resistance (IR). We aimed to clarify the safety and/or the protective potential of CrPic_3_ on Leydig cell steroidogenesis and antioxidant balance. To achieve these goals, we assessed CrPic_3_ cytotoxicity in BLTK1 LCs in IR conditions. We further examined the impact of CrPic_3_ on LC steroidogenesis, analyzed the exometabolome of CrPic_3_-exposed cells, and evaluated the mitochondrial function post-CrPic_3_ exposure in the IR LC model. We further investigated CrPic_3_ possible protective effects against oxidative stress, focusing on protein and lipid damage in the IR LC model.

## 2. Materials and Methods

### 2.1. Chemicals

Dulbecco’s modified Eagle medium–low glucose Ham’s Nutrient Mixture F12 with L-glutamate and without sodium bicarbonate (DMEM: HAM’s F12), fetal bovine serum (FBS), sodium dodecyl sulfate (SDS), β-mercaptoetanol, 2,4-dinitrophenylhydrazine (DNPH), and trypsin-ethylenediaminetetraacetic acid (EDTA) were purchased from Sigma–Aldrich (St. Louis, MO, USA). Insulin–transferrin–selenium (ITS), trifluoracetic acid (TFA), and penicillin and streptomycin (Pen-Strep) were obtained from Gibco | ThermoFisher Scientific (Waltham, MA, USA). Gentamicin was obtained from lonza (Basel, Switzerland). Sulforhodamine B (SRB) was purchased from Biotium (Hayward, CA, USA), while 3-(4,5-dimethylthiazol-2-yl)-2,5-diphenyltetrazolium bromide (MTT) was obtained from Amresco (Solon, OH, USA) and the lactate dehydrogenase (LDH)-CytoxTM Assay Kit was obtained from BioLegend^®^ (San Diego, CA, USA). The Triton-x 100, 0.5% Ponceau Solution, dimethyl sulfoxide (DMSO), amphotericin B, and palmitate (PA) were all obtained from Merck-Sigma-Aldrich (Darmstadt, Germany). CrPic_3_ was purchased from Chemscene (Monmouth Junction, New Jersey USA). Phenylmethanesulfonyl fluoride (PMSF) and sodium ortho-vanadate were purchased from AppliChem (Darmstadt, Germany). A protease inhibitor cocktail (AEBSF, Aprotinin, Bestatin, E-64, Leupeptin, and Pepstatin A) was acquired from Bimake (Huissen, The Netherlands). Bovine serum albumin (BSA) and Tris base were purchased from NZYTech (Lisbon, Portugal). A Clarity Western Enhanced Chemiluminescence (ECL) substrate kit and a 12.5% polyacrylamide gel kit TGX Stain-Free™™ FastCast™™ Acrylamide Kit were acquired from Bio-Rad (Hemel Hempstead, UK). An ELISA (enzyme-linked immunosorbent assay) Kit was obtained from FineTest (Boulder, CO, USA).

### 2.2. Cell Culture—BLTK1 Murine Leydig Cells

BLTK1 is a murine LC line first established in 1996 with the name BLT-1. This cell line was immortalized from a testicular tumor induced by the Simian virus T-antigen (SV40 TAG) oncogene of transgenic mice expressing the inhibin-α promoter [35]. These cells resemble other LC lines, such as MA-10 and mLTC-1, and respond to hCG signaling by stimulating the production of cAMP and progesterone but exhibit low testosterone production [36]. In 2018, it was revealed that this low testosterone production is justified by altered steroidogenic pathways, in which 17β-HSD is barely expressed. Thus, the only way to accurately study the production of testosterone by LCs is to use human or mouse primary LCs [37]. However, LCs can be used in studies of the endocrine, paracrine, and autocrine regulation of LCs and how these can be disrupted by EDCs [36,38]. Furthermore, it might be interesting to access steroidogenesis by measuring intermediates of this process, such as androstenedione. The cells were kindly provided by Nafis Rahman, MD, Ph.D., Faculty of Medicine, Institute of Biomedicine, University of Turku (Finland); cultured in 75 cm^2^ T-flasks; and kept at 37 °C with a 5% (*v*/*v*) CO_2_ humified atmosphere in culture medium DMEM:Ham’s F12 1:1 supplemented with 10% heat-inactivated FBS, 50 μm/mL gentamicin, 1% Pen-Strep, 1% amphotericin B (50 U/mL penicillin, 50 mg/mL streptomycin sulfate, and 0.5 mg/mL fungizone), 15 mM HEPES, and 14 mM NaHCO_3_. Cells from passages 15 to 25 were used, and their morphology and functionality were routinely assessed via optical microscopy and androgen quantification, respectively.

### 2.3. Experimental Groups and Design

**Figure 1:**
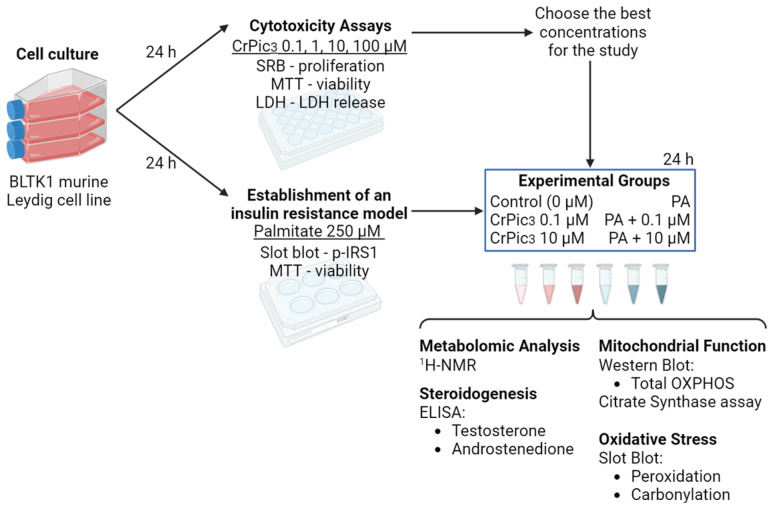
Study design involving Leydig cell culture, cytotoxicity assays, establishment of an insulin resistance model, and evaluation of physiological parameters such as exometabolome, mitochondrial parameters, oxidative stress, and androgen production.

This study included an initial phase of cell culture, which was followed by exposure to CrPic_3_ for 24 h, after which cytotoxicity assays were performed. At the same time, an in vitro model of IR was established by exposing the cells to PA. Using this in vitro model, several parameters of the LC physiology were assessed, namely its exometabolome, mitochondrial parameters, oxidative stress, and androgen production (Figure 1).

For the cytotoxicity evaluation, CrPic_3_ was diluted in DMSO to achieve stock concentrations of 0 µM (control), 0.1 µM, 1 µM, 10 µM, and 100 µM, which were used to expose the seeded cells when the desired confluency was reached. These concentrations were chosen considering that the supplements commercially available lead to absorptions from 3 μg to 60 μg. Hence, the concentrations of CrPic_3_ used in this study, chosen to resemble chronic accumulation, were 0.1 μM and 1 μM to represent lower testicular accumulation and 10 μM and 100 μM to represent higher testicular accumulation. Also, each treatment included the control group composed of the culture medium previously described, with no CrPic_3_ and the addition of the same concentration of DMSO as the other groups (0.5%). Moreover, the standardized duration of each treatment was 24 h.

Regarding the experimental groups established to investigate the potential protective effects of CrPic_3_ on LCs affected by IR, concentrations of 0.1 µM and 10 µM were chosen. To induce IR, PA was used at a concentration of 25 mM, dissolved in 50 mM of NaOH at 70 °C, and mixed with 10% (*w*/*v*) bovine serum albumin (BSA) at 55 °C for a final concentration of 250 µM, as described by Qin and colleagues [39]. For this, to avoid eventual residual levels of PA on FBS, ITS was used as a substitute for FBS in the culture medium. Thus, for this phase of the laboratory work, the following six treatment groups were studied: control, 0.1 µM of CrPic_3_ (and 0 µM of PA), 10 µM of CrPic_3_ (and 0 µM of PA), 0 µM of CrPic_3_ and 250 µM of PA, 0.1 µM of CrPic_3_ and 250 µM of PA, and 10 µM of CrPic_3_ and 250 µM of PA. Similarly, the duration of the treatments was 24 h.

### 2.4. Evaluation of Cytotoxic Profile of the CrPic_3_ and PA

#### 2.4.1. Sulforhodamine B Cytotoxicity Assay

For the SRB assay, cells were seeded in 24-well plates and exposed to CrPic_3_ when a confluency of approximately 60% was reached. After 24 h, after which the treatment medium was removed, cells were washed using PBS and fixed with a fixation solution (1% acetic acid in 99% of methanol) for at least 1 h at −20 °C. Then, cells were incubated with a solution of 0.05% SRB prepared with 1% acetic acid in water at 37 °C. After 1 h of incubation, cells were again washed with 1% acetic acid in water. Using a Tris solution with pH 10, the SRB dye bound to the proteins of the BLTK1 cells was removed, and 100 μL of the resulting solution was transferred to a 96-well plate in duplicates. The optical density of these solutions was determined at 510 nm using a multiplate reader (MultiSkan Go, Thermo Fisher Scientific, Vantaa, Finland). The obtained results were divided by the mean of the control group and expressed in fold variation to the control.

#### 2.4.2. Lactate Dehydrogenase Release Assay

To perform this assay, BLTK1 cells were cultured in 24-well plates and exposed to CrPic_3_ concentrations for 24 h. Then, 100 μL was transferred from each well to a 96-well plate in duplicates. An aliquot of 100 μL of the LDH assay buffer from the kit LDH-CytoxTM Assay Kit (BioLegend^®^, San Diego, CA, USA) was added to each well, and the plate was incubated in the dark for 30 min at 37 °C, as instructed by the manufacturer. Then, the reaction was interrupted with 50 μL of the stop solution, and the optical density of each well was determined at 490 nm using a multiplate reader (MultiSkan Go, Thermo Fisher Scientific, Vantaa, Finland). The obtained results were divided by the mean of the control group and expressed in fold variation to the control.

#### 2.4.3. 3-(4,5-Dimethylthiazol-2-yl)-2,5-Diphenyltetrazolium Bromide Assay

Our experimental design included two moments when the MTT assay was performed. The first was to assess the possible cytotoxic effects of CrPic_3_, and the second was to examine the cytotoxicity of PA to establish an in vitro model of IR in LCs. In both cases, cells were seeded and exposed in a 48-well plate for 24 h with the respective doses. After the treatment, the medium was removed, replaced with 500 µL DMEM:Ham’s F12 medium and 50 µL of MTT solution at 5 mg/mL, and incubated for 2 h at 37 °C. Following incubation, this medium was removed, 250 µL of DMSO was added to solubilize formazan, and from this solution, 50 µL was transferred to a 96-well plate and diluted in 50 µL of DMSO. The absorbances were measured at 570 nm and 655 nm using a multiplate reader (MultiSkan Go, Thermo Fisher Scientific, Vantaa, Finland). For the statistical analysis, absorbances measured at 655 nm were subtracted from the ones measured at 570 nm. The resulting values were divided by the mean of the control group and expressed in fold variation to the control.

### 2.5. Exometabolome Analysis via ^1^H-NMR Spectroscopy

LCs were exposed to CrPic_3_ and PA in 75 cm^2^ T-flasks for 24 h, after which the culture medium was collected for exometabolome profiling, and the cells were detached from the flask by using 0.05% trypsin-EDTA for protein extraction. The protein fraction was used for Western blot, slot-blot, and a citrate synthase (CS) assay. For the exometabolome profiling of LCs exposed to CrPic_3_ and PA, the ^1^H-NMR spectra of the medium of each group were acquired using 180 μL of culture medium collected from the flasks, using the methods described by Alves and colleagues [40]. In brief, the ^1^H-NMR spectra were acquired using a 500 MHz Bruker Avance III HD spectrometer equipped with a 5 mm TXI probe (Bruker Corporation, Billerica, MA, USA), working at 298 K. Solvent-suppressed 1D-1H-NMR spectra were acquired using a zgpr pulse sequence, with a sweep width of 7 kHz, relaxation delay of 7 s, pulse angle of 30°, acquisition time of 2.3 s, and 64 scans. For sample analysis, 225 μL of a mixture containing 10 mM of sodium fumarate with 180 μL of culture medium was transferred to an NMR tube and analyzed. Spectra were processed by applying a line broadening of 0.2 Hz before Fourier transformation and manually phased and baseline-corrected. Sodium fumarate (6.50 ppm) was used as an internal reference for chemical shifts and metabolite quantification. The NUTS-Pro NMR software (Acorn NMR, Inc, Fremont, CA, USA) was used for the processing of the spectra and the quantification of the following metabolites (multiplicity, chemical shift): sodium fumarate (final concentration of 2 mM) used as an internal reference (6.50 ppm), lactate (doublet, 1.33), alanine (doublet, 1.45), pyruvate (singlet, 2.34), glutamine (triplet, 3.77), and H1-α-glucose (doublet, 5.22). Results are expressed as metabolite variation (consumption or production) in μmol/mg protein.

### 2.6. Protein Extraction and Quantification

Total protein was extracted from LCs using Triton-X buffer (Triton-X 100, HEPES 10 Mm, EGTA 0.5 mM), which was supplemented with a lysis buffer composed of 100 mM PMSF, 1% protease inhibitor cocktail, and 100 mM sodium orthovanadate. A total of 50 μL of this lysis buffer was added to the cell homogenate, and the resulting solution was agitated for 10 min (room temperature). Total protein quantification was performed using the PierceTM Microplate BCA Protein Assay Kit according to the instructions of the manufacturer. The calibration curve was calculated by using different concentrations of BSA as standards for protein quantification. Sample absorbances were measured at 560 nm using a multiplate reader (MultiSkan Go, Thermo Fisher Scientific, Vantaa, Finland).

### 2.7. Western Blot

For this technique, 75 μg of total protein from each sample was mixed with an adequate volume of sample buffer (62.5 mM Tris-HCl, pH 6.8, 25% glycerol, 2% SDS, and 0.01% bromophenol blue, with 5% β-mercaptoethanol). Samples with the sample buffer were denatured for 10 min at 37 °C. Proteins were fractionated using the TGX Stain-Free FastCast Acrylamide kit with 15% polyacrylamide gel, as per the manufacturers’ instructions, and electrophoresis was carried out at 90 V until the running front reached the desired height. Then, proteins were electrotransferred from the gel into a polyvinylidene difluoride (PVDF) membrane with a Trans-Blot^®^ Turbo^TM^ Transfer System (Bio-Rad, Hemel Hempstead, United Kingdom), using the preprogrammed protocol mixed molecular weight (7 min at 25 V and 1.3 mA per gel). The total loaded protein was measured using TGX Stain-Free fluorescence imaging, as described by the supplier (Bio-Rad, Hemel Hempstead, United Kingdom), which eliminates the need for a housekeeping protein control that is often found to be erroneously used in the literature, as explained by Maloy and colleagues [41], and computed with Image Lab software (BioRad, Hemel Hempstead, United Kingdom). Subsequently, membranes were blocked for 3 h in a 5% non-fat milk dissolved in washing buffer (1.54 M NaCl, 1 M Tris-Base, pH 8.0 and Tween 20) at room temperature. Then, membranes were incubated overnight at 4 °C with the primary Antibody Cocktail MitoProfile^®^ Total OXPHOS dissolved in 1% non-fat milk (Table 1). On the following day, membranes were washed 3 times with washing buffer and incubated with the corresponding secondary antibody for 90 min at room temperature. Then, membranes were washed again and incubated with the reagent from the Clarity Western ECL substrate (3 mL per membrane) for 5 min, as instructed by the manufacturer. Then, the membranes were read with the Bio-Rad ChemiDoc XRS. For the statistical analysis, bands corresponding to the protein of interest were quantified using Image Lab^TM^ standard procedures. Each band density was divided by the respective total protein density using the BioRad TGX gel kit and normalized with the control group value, thus being expressed as fold variation to the control group.

### 2.8. Slot-Blot

Slot-blot was performed to assess the individual levels of phosphorylated IRS-1 (p-IRS-1) on serine 307 (Ser^307^) and hallmarks of oxidative stress. The chosen hallmarks were lipid peroxidation and protein carbonylation, and, for this, we focused on identifying the products of these reactions, such as 4-hydroxynonenal (4-HNE) for peroxidation and 2,4-dinitrophenyl (DNP) for carbonylation [42]. Samples to be used for carbonylation were firstly derivatized using DNPH to DNP. To derivatize these samples, 10 µL of the cell protein extract was diluted in filtered PBS up to a final volume of 50 µL. Then, 50 µL of 12% SDS was added. After homogenization, 100 µL of 20 mM DNPH (Sigma-Aldrich, USA) in 10% TFA was added to the oxidative stress hallmarks solution, after which these samples were incubated at room temperature in a light-protected environment. After 30 min, 7.5 µL of 18% β-mercaptoetanol in Tris 2 M was added to the mixture. From this point, the derivatized samples for carbonylation and the samples for p-IRS-1 and peroxidation were diluted in PBS to a concentration of 0.01 µg/mL. Afterward, 100 µL of these diluted solutions were transferred to a PVDF membrane (Amersham™ Protran™, GE Healthcare, Munich, Germany, 0.45 μm porosity) using a slot-blot system (Hybri-SlotTM Manifold, Bethesda Research Laboratories, Bethesda, MD, USA). Then, membranes were blocked for 90 min in 5% non-fat milk and incubated overnight at 4 °C with the appropriate antibody (Table 1). After 90 min of incubation with the secondary antibody, membranes were incubated with a Bio-Rad Clarity ECL substrate and read using Bio-Rad ChemiDoc XRS (Bio-Rad, Hemel Hempstead, UK). Densities from each band were quantified using Image LabTM standard procedures, and the results were expressed as fold variation to the control group.

### 2.9. Citrate Synthase Activity Assay

The citrate synthase (CS) activity was evaluated in the LC homogenates by using the procedure previously described [43]. In brief, CS is the enzyme that catalyzes the reaction of acetyl-CoA with oxaloacetate to form citrate, with the release of coenzyme A (CoA). In this assay, the free thiol group of CoA reacts with 5,5′-dithiobis-(2-nitrobenzoic acid). This reaction causes the formation of the anion 2-nitro-5-thiolbenzoate (molar absorptivity constant of 13.6 mM^−1^·cm^−1^), which has an absorption peak around 412 nm. The absorbance was read for approximately 2 min at 30 °C in a microplate reader (Multiskan GO, Thermo Fischer Scientific, Northumberland, UK). CS activity was calculated by dividing the obtained value by the molar absorptivity constant, which was multiplied by the ratio between the final and sample volumes and normalized to total protein.

### 2.10. Competitive ELISA

Since the BLTK1 cells, as happens with other LC lines, do not functionally express 17β-HSD_3_, it is not possible to directly measure the testosterone production, since these produce little to no testosterone under basal conditions [37]. Therefore, to assess steroidogenesis, a competitive ELISA kit was used to detect androstenedione levels, the intermediate androgen that is reduced to testosterone by 17β-HSD_3_. To facilitate quantification, 1 mL of the culture medium of BLTK1 cells exposed to CrPic_3_ and PA for 24 h was concentrated using the SpeedVac Concentrator (ThermoFisher Scientific, North Carolina, USA) and resuspend in 200 µL of DMEM: HAM’s F12. To quantify androstenedione, the instructions of the manufacturer were followed. In brief, the assay plate was washed 2 times, and 50 µL of the concentrated culture medium of BLTK1 was added to the plate. A total of 50 µL of a biotin-labeled antibody working solution was also added, with static incubation for 45 min at 37 °C. After this, the well content was removed, and each well was washed 3 times. Then, 100 µL of an HRP-streptavidin conjugate was added to each well, and the plate was incubated for 30 min at 37 °C. The content of the well was removed and washed 5 times, and 90 µL of the TMB (3,3′,5,5′-tetrametilbenzidina) substrate was added to the wells and incubated for 15 min, after which the optical density was measured at 450 nm using a multiplate reader (MultiSkan Go, Thermo Fisher Scientific, Vantaa, Finland). The calibration curve was calculated by using different concentrations of androstenedione as standards for quantification (10, 5, 2.52, 1.25, 0.625, 0.312, and 0.156 ng/mL), and the concentration of androstenedione in each well was from the obtained absorbances using the software CurveExpert (FineTest, USA). The production of androstenedione was calculated by dividing the obtained result by the respective dilution factor and expressed in ng/mL.

### 2.11. Statistical Analysis

In the text, the data are presented as mean ± standard error of the mean (SEM) of replicates of *n* = 6 to *n* = 8, depending on the procedure. In the figures, the data are presented as box plots from minimum to maximum, where the line represents the median. Statistical significance was assessed by using one-way ANOVA and GraphPad Prism 8 (GraphPad software, Boston, Massachusetts, USA), with the significance level set at 5% (*p*-value < 0.05).

## 3. Results

### 3.1. High Doses of CrPic_3_ Are Cytotoxic, While Lower Doses Do Not Cause Significant Damage to LCs

**Figure 2:**
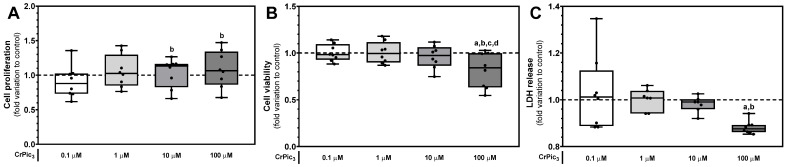
Evaluation of the cytotoxic profile of different concentrations of chromium picolinate (CrPic_3_) on LCs through (**A**) SRB assay for cellular proliferation (*n* = 8 for each condition), (**B**) MTT assay for cell metabolic viability (*n* = 8 for each condition), and (**C**) LDH release assay for cell membrane damage (*n* = 8 for each condition). Significantly different results (*p* < 0.05) are indicated above one of the groups as a—relative to the control (without CrPic_3_)), b—relative to 0.1 µM CrPic_3_, c—relative to 1 µM CrPic_3_, and d—relative to 10 µM CrPic_3_. Control is represented by the dashed line. Abbreviations: LDH—lactate dehydrogenase; MTT—3-(4,5-dimethylthiazol-2-yl)-2,5-diphenyltetrazolium bromide; SRB—Sulforhodamine B.

When evaluating cellular proliferation using the SRB assay, we observed a significant increase when LCs were exposed to concentrations of 10 µM (1.03 ± 0.07-fold variation to control) and 100 µM (1.09 ± 0.10-fold variation to control) of CrPic_3_ when compared to those exposed to 0.1 µM (0.91 ± 0.08-fold variation to control). No significant differences were found between the proliferation of the LCs of any of the experimental groups when compared to those in the control group (1.00 ± 0.01-fold variation to control) (Figure 2A). Concerning the evaluation of the metabolic viability, using the MTT assay, the LCs exposed to the highest concentration of CrPic_3_ (100 µM) showed a reduction in the cell metabolic viability, since a significant difference was found between the group of LCs exposed to 100 µM of CrPic_3_ (0.82 ± 0.07-fold variation to control) when compared to that of cells from other groups, namely from the control group (1.00 ± 0.01-fold variation to control) and from the groups exposed to 0.1 µM (1.00 ± 0.03-fold variation to control), 1 µM (1.00 ± 0.04-fold variation to control), and 10 µM (0.96 ± 0.04-fold variation to control) of CrPic_3_ (Figure 2B). Finally, the data obtained from the LDH release assay showed that the cells exposed to 100 µM of CrPic_3_ (0.88 ± 0.01-fold variation to control) had significantly lower LDH release to the extracellular medium when compared to the LCs from the control group (1.00 ± 0.01-fold variation to control) and from the group exposed to 0.1 µM (1.03 ± 0.06-fold variation to control) of CrPic_3_ (Figure 2C).

### 3.2. An In Vitro Model of Insulin Resistance Was Successfully Established in LCs, and CrPic_3_ Was Not Able to Revert This Condition

**Figure 3:**
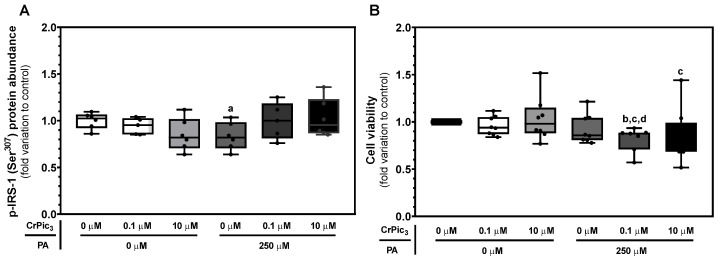
Establishment of the in vitro model of insulin resistance on LCs and assessment of the effects of chromium picolinate (CrPic_3_). (**A**): Quantification of protein levels of phosphorylated insulin receptor substrate 1 (p-IRS-1) on serine 307 (Ser307) in LCs exposed to CrPic_3_ (0, 0.1, and 10 μM) and palmitate (PA) (0 and 250 μM). (**B**): Evaluation of cytotoxic effects on LC metabolic viability of CrPic_3_ and PA (*n* = 8 for each condition). Significantly different results (*p* < 0.05) are indicated above one of the groups as a—relative to the control (without CrPic_3_ and PA), b—relative to 0.1 μM CrPic_3_ without PA, c—relative to 10 μM CrPic_3_ without PA, and d—relative to 250 μM PA without CrPic_3_.

**Figure A1:**
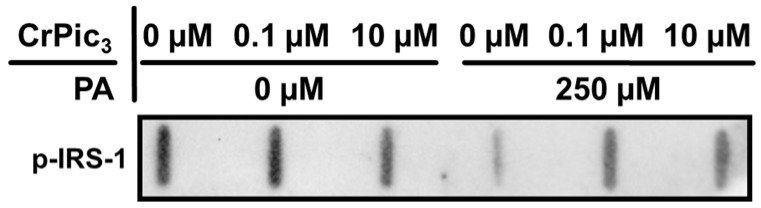
Representative image of the slot-blot analysis regarding the effects of chromium picolinate (CrPic_3_) and palmitate (PA) in the relative levels of phosphorylated Insulin receptor substrate 1 (p-IRS-1) to assess the insulin pathway to validate the in vitro insulin resistance model in BLTK1 cells.

To assess whether the insulin resistance in vitro model was successfully induced by PA, an immunoblot was performed using an anti-p-IRS-1 (Ser^307^) antibody (Figure 3A). The LCs exposed to 250 µM of PA (0.83 ± 0.06-fold variation to control) showed a reduction in p-IRS-1 (Ser^307^) abundance when compared to the cells from the control group (1.00 ± 0.03-fold variation to control). The concentrations of CrPic_3_ used to assess if it can revert the effects of PA were 0.1 µM and 10 µM. Hence, the experimental groups of the remaining experiments consisted of a control group (0 µM of CrPic_3_ and PA), two groups only with the CrPic_3_ concentrations, a PA-only group, and two groups with CrPic_3_ and PA. CrPic_3_-exposed LC did not show an increase in p-IRS-1 (Ser^307^), both when it was administered alone or in the presence of PA (insulin resistance model). A representative image of the immunoblots obtained for this protein can be found in Figure A1 in Appendix A. To determine the cytotoxic effects of PA in LCs, the MTT assay for cell metabolic viability was used, with a significant decrease being identified between the LCs exposed to 0.1 µM of CrPic_3_ with PA (0.81 ± 0.049-fold variation to control) and the cells exposed to just 0.1 µM of CrPic_3_ (0.96 ± 0.035-fold variation to control), 10 µM of CrPic_3_ (1.03 ± 0.08-fold variation to the control), and PA (0.93 ± 0.05-fold variation to control). A significant decrease was also observed in the LCs exposed to 10 µM of CrPic_3_ in combination with PA (0.84 ± 0.10-fold variation to the control) when compared to the cells only exposed to 10 µM of CrPic_3_ (Figure 3B).

### 3.3. Exometabolome Profiling of LC Exposed to CrPic_3_ and PA

#### 3.3.1. CrPic_3_ Exposure Did Not Alter Glucose or Glutamine Consumption in LCs under IR Conditions

**Figure 4:**
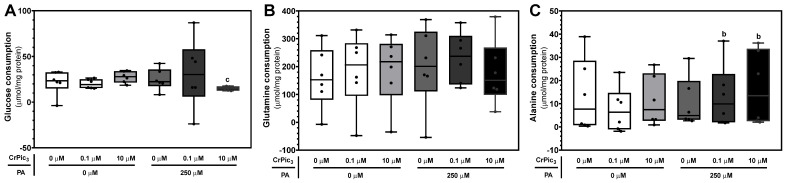
Consumption of glucose (**A**), glutamine (**B**), and alanine (**C**) by LCs after treatment with chromium picolinate (CrPic_3_) and/or palmitate (PA). The metabolites were measured through ^1^H-NMR analysis of the extracellular medium after 24 h incubation. Results are expressed as micromole per milligram of protein and as mean ± SEM (*n* = 6 for each condition). Significantly different results (*p* < 0.05) are indicated above one of the groups as b—relative to 0.1 µM CrPic_3_ without PA and c—relative to 10 µM CrPic_3_ without PA.

In the present work, LCs exposed to 10 µM of CrPic_3_ plus 250 µM of PA (15.1 ± 1.06 μmol/mg protein) showed a significant decrease in glucose consumption when compared to the cells exposed only to 10 µM of CrPic_3_ (27.4 ± 2.56 μmol/mg protein), with no differences compared to the control (Figure 4A). No alterations were observed in the consumption of glucose of the LCs exposed only to CrPic_3_, only PA, or CrPic_3_ 0.1 µM plus PA. In our study, we further observed the consumption of glutamine and alanine by LCs. No significant differences were observed between the LCs of the groups exposed to CrPic_3_ and/or PA regarding the consumption of glutamine (Figure 4B), with cells from the control group consuming 160 ± 45 μmol/mg protein of glutamine. Alanine consumption, on the other hand, was found to be significantly increased in the groups of LCs exposed to PA plus CrPic_3_ 0.1 µM (13.1 ± 5.48 μmol/mg protein) and 10 µM (16.8 ± 6.47 μmol/mg protein), when compared to the LCs of the group exposed only to 0.1 µM of CrPic_3_ (7.53 ± 3.95 μmol/mg protein) (Figure 4C).

#### 3.3.2. CrPic_3_ Did Not Alter the Production of Pyruvate or Lactate by LCs Even in IR Conditions

**Figure 5:**
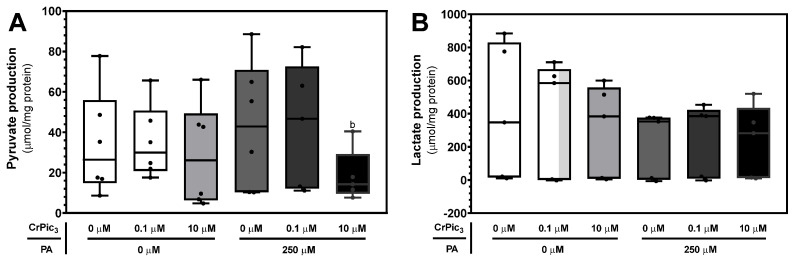
Production of pyruvate (**A**) and lactate (**B**) by LCs after treatment with chromium picolinate (CrPic_3_) and/or palmitate (PA). The metabolites were measured through ^1^H-NMR analysis of the extracellular medium after 24 h incubation. Results are expressed as micromole per milligram of protein and as mean ± SEM (*n* = 6 for each condition). Significantly different results (*p* < 0.05) are indicated above one of the groups as b—relative to 0.1 µM CrPic_3_ without PA.

In the present work, the group of LCs exposed to 10 µM of CrPic_3_ and PA (18.4 ± 5.8 μmol/mg protein) had significantly lower production of pyruvate than that of LCs exposed to 0.1 µM of CrPic_3_ (35.1 ± 7.4 μmol/mg protein), with no differences between any of the cells from these groups and those of the control group (34.1 ± 10.5 μmol/mg protein) (Figure 5A). Finally, no significant differences were found between the cells from the various experimental groups concerning lactate production (Figure 5B). For this metabolite, LCs from the control group exhibited a production of 407.4 ± 183.3 μmol/mg protein.

It is important to note that in the reaction catalyzed by LDH, one molecule of lactate is produced from each pyruvate molecule [44], which means that a molecule of glucose is able to produce two molecules of lactate. When comparing the production of both metabolites, it can be noted that the production of lactate by LCs of the different experimental groups is 5 to 12 times higher than that of pyruvate. Table 2 depicts the ratios of carbon equivalents for the LCs from the various experimental groups considering the production and consumption pathways of the metabolites measured in the culture media. 

### 3.4. Exposure of LCs to CrPic_3_ Reduces the Expression of Mitochondrial Complexes Associated with Oxidative Stress and Reduces Oxidative Damage to Lipids Caused by IR

**Figure 6:**
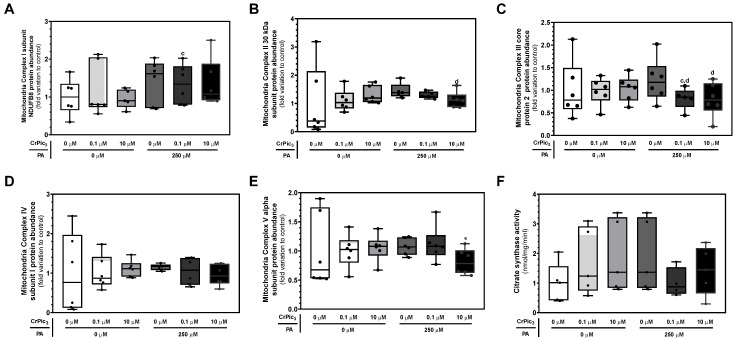
Assessment of the mitochondrial parameters of LCs exposed to chromium picolinate (CrPic_3_) and palmitate (PA). (**A**–**E**) Western blot analysis of mitochondrial complexes involved in OXPHOS after exposure of LCs to CrPic_3_ and PA via quantification of (**A**) complex I subunit NDUFB8, (**B**) complex II 30 kDa subunit, (**C**) complex III Core protein 2, (**D**) complex IV subunit I, and (**E**) complex V alpha subunit. Results are expressed as mean ± SEM (*n* = 6 for each condition) and presented as fold variation to the control group. Significantly different results (*p* < 0.05) are indicated above one of the groups as c—relative to 10 μM CrPic_3_ without PA, d—relative to 250 µM PA without CrPic_3_, and e—relative to 0.1 µM CrPic_3_ plus 250 µM PA. (**F**) Citrate synthase activity assay to assess the impact of CrPic_3_ and/or PA on mitochondria content of LCs. The results are expressed as mean ± SEM (*n* = 6 for each condition) and presented as nmol/min/mg. No significant results were found.

**Figure A2:**
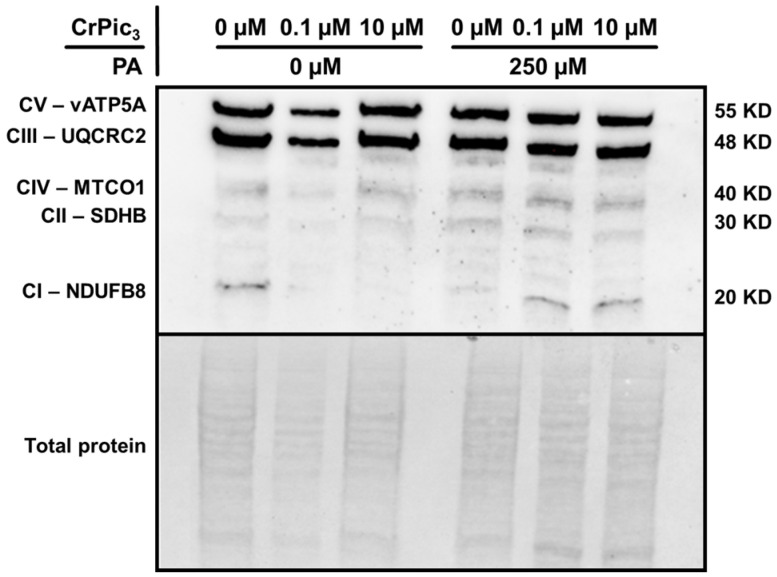
Representative image of the Western blot analysis regarding the effects of chromium picolinate (CrPic_3_) and palmitate (PA) in the relative levels of the five OXPHOS complexes of the mitochondrial membrane respiratory chain. These complexes include Complex I or NADH dehydrogenase (CI—NDUFB8), Complex II or succinate dehydrogenase (CII—SDHB), Complex III or ubiquinol-cytochrome c reductase (CIII—UQCRC2), Complex IV or cytochrome c oxidase (CIV—MTCO1), and Complex V or F_1_F_o_ ATP synthase (CV—vATP5A), with molecular weights of 20 KD, 30 KD, 48KD, 40 KD, and 55 KD, respectively. Bands representative of each complex and total protein are presented.

After exposure to CrPic_3_ (0.1 µM or 10 µM) and/or PA (250 µM), we performed an evaluation of the levels of the mitochondrial complexes to assess whether CrPic_3_ and/or PA affect mitochondrial oxidative function through the modulation of the electron transport chain complexes of the mitochondria of LCs. Concerning the subunit NDUFB8 of mitochondrial complex I, LCs exposed to 0.1 µM CrPic_3_ and PA (1.34 ± 0.21-fold variation to the control) showed a significant increase in the levels of this complex compared to LCs exposed to 10 µM CrPic_3_ (0.95 ± 0.11-fold variation to the control) (Figure 6A). As for the 30 kDa subunit of mitochondrial complex II, the group of LCs exposed only to PA (1.44 ± 0.12-fold variation to the control) showed significantly higher levels of this complex than that of LCs exposed to PA and 10 µM CrPic_3_ (1.13 ± 0.11-fold variation to the control) (Figure 6B). A similar trend was observed for the core protein 2 subunit of mitochondrial complex III, with the group of cells exposed to 0.1 µM CrPic_3_ plus PA (0.82 ± 0.11-fold variation to control) and CrPic_3_ 10 µM plus PA (0.80 ± 0.15-fold variation to the control) showing significantly lower levels of this complex than the cells exposed to PA solely (1.22 ± 0.19-fold variation to the control) (Figure 6C). None of the groups of BLTK1 cells exhibited statistical differences regarding the abundance of subunit I of mitochondrial complex IV (Figure 6D). Finally, the cells exposed to 10 µM CrPic_3_ and PA (0.81 ± 0.09-fold variation to the control) were found to have a significantly lower expression of the alpha subunit of complex V than the cells exposed to 0.1 µM CrPic_3_ and PA (1.12 ± 0.12-fold variation to the control) (Figure 6E). A representative image of the blots obtained can be found in Figure A2 in Appendix A. To further characterize the effects of CrPic_3_ in LC mitochondria and elucidate if it was able to protect them from the impact of exposure to PA, the activity of the mitochondrial enzyme CS was assessed by using a spectrophotometric kinetic enzyme assay since this enzyme is considered a marker of the mitochondria content in cells [45]. This assay showed no significant differences in the enzyme activity between the groups exposed to CrPic_3_ and/or PA and the control group (Figure 6F).

**Figure 7:**
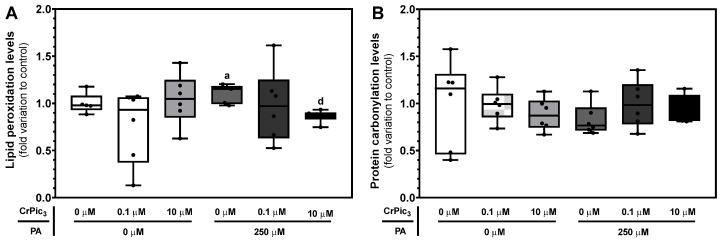
Oxidative hallmark profiling of lipid peroxidation (**A**) and protein carbonylation (**B**) of LCs after exposure to experimental groups with chromium picolinate (CrPic_3_) and/or palmitate (PA), assessed by slot-blot. The results are expressed as mean ± SEM (*n* = 6 for each condition) and presented as fold variation to the control group. Significantly different results (*p* < 0.05) are indicated as a—relative to the control (without CrPic_3_ and PA) and d—relative to 250 µM PA without CrPic_3_.

**Figure A3:**
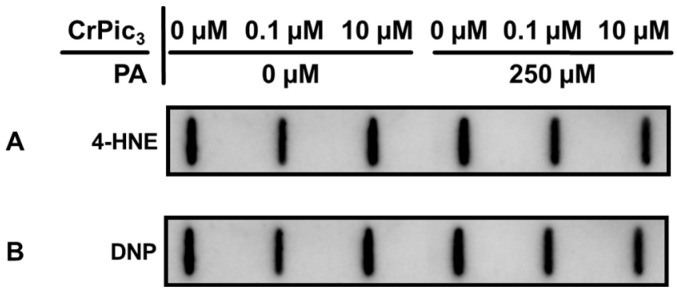
Representative image of the slot-blot analysis regarding the effects of chromium picolinate (CrPic_3_) and palmitate (PA) in the relative levels of oxidative stress hallmarks. (**A**) 4-hydroxynonenal (4-HNE) for peroxidation and (**B**) 2,4-dinitrophenyl (DNP) for carbonylation.

To evaluate the oxidative status of LCs after exposure to CrPic_3_ (0.1 µM or 10 µM) and/or PA (250 µM), the slot-blot technique was used to assess lipid peroxidation (Figure 7A) and protein carbonylation (Figure 7B), hallmarks of cellular oxidative stress. Concerning lipid peroxidation, the group of LCs exposed only to PA (1.10 ± 0.04-fold variation to the control) showed a significant increase in lipid peroxidation levels compared to the LCs of the control group (1.00 ± 0.05-fold variation to the control), whereas the group of LCs exposed to a combination of 10 µM CrPic_3_ plus PA (0.86 ± 0.03-fold variation to the control) showed a decrease in this oxidative stress hallmark to levels similar to the control group. In the case of protein carbonylation, no significant differences were detected when comparing the cells from the various groups to those of the control. A representative image of the blots obtained via slot-blot of this protein can be found in Figure A3 in Appendix A.

### 3.5. LC Exposure to CrPic_3_ Only Increases Androstenedione Production in the Presence of PA

**Figure 8:**
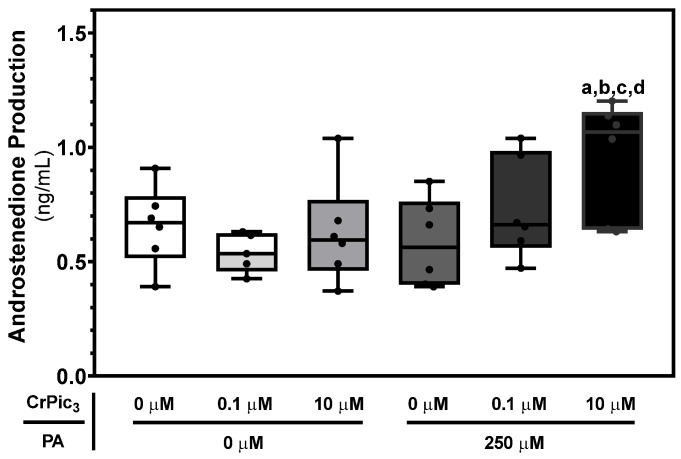
Androstenedione secretion by LCs after exposure to chromium picolinate (CrPic_3_) and/or palmitate (PA) assessed using ELISA (enzyme-linked immunosorbent assay). The results are expressed as mean ± SEM (*n* = 6 for each condition) and presented in ng/mL. Significantly different results (*p* < 0.05) are indicated as a—relative to the control (without CrPic_3_ and PA), b—relative to 0.1 µM CrPic_3_ without PA, c—relative to 10 µM CrPic_3_ without PA, and d—relative to 250 µM PA without CrPic_3_.

Since the main function of LCs is to produce testosterone via steroidogenesis [46], any study regarding the effect of a substance in these cells should focus on this primary function. Hence, we used an ELISA kit to measure basal androstenedione production, as it is the final product of steroidogenesis in these cells, as previously mentioned [37]. The group of LCs exposed to 10 µM CrPic_3_ and PA (0.96 ± 0.10 ng/mL) showed significantly higher androstenedione production than the LCs from the control group (0.65 ± 0.07 ng/mL) and from the groups of cells exposed to 0.1 µM of CrPic_3_ (0.54 ± 0.03 ng/mL), 10 µM of CrPic_3_ (0.62 ± 0.09 ng/mL), and 250 µM of PA (0.58 ± 0.08 ng/mL) (Figure 8).

## 4. Discussion

Almost sixty years ago, trivalent chromium (Cr(III)) was designated as an essential metal [47]. However, this classification has been a topic of debate, primarily because of safety issues [18,48,49]. Chromium picolinate (CrPic_3_) is the most commonly used Cr(III) compound, often associated with its potential role in managing blood glucose levels [20] and decreasing damage caused by oxidative stress [25,26,27,28]. This ongoing discussion has led to concerns about the safety and effectiveness of its continued use. Notably, the existing literature lacks comprehensive pharmacokinetic models that elucidate how CrPic_3_ is processed and accumulates in the testes and in individual testicular cells (e.g., Leydig cells), along with its potential impact.

To investigate the influence of CrPic_3_ on LCs, we assessed its cytotoxicity in LCs. Interestingly, none of the concentrations chosen altered cell proliferation. However, higher concentrations led to increased proliferation. This increase could be interpreted as a beneficial effect or a compensatory mechanism to counteract potential adverse effects, although such a mechanism has not been previously described. Furthermore, the application of 100 μM CrPic_3_ resulted in decreased metabolic viability, even though it protects against membrane damage. These findings indicate that high concentrations of CrPic_3_ have a detrimental impact on LCs, affecting the viability of the cells. Consequently, we excluded the 100 μM concentration from subsequent experiments. Additionally, the 1 μM concentration was omitted as it did not produce significant differences in cell proliferation compared to groups exposed to 0.1 μM and 10 μM of CrPic_3_. Hence, we used 0.1 and 10 μM concentrations of CrPic_3_ for the subsequent stages of our study.

Considering the distinct antidiabetic properties of CrPic_3_, it was deemed essential to investigate its impact on LCs under conditions of IR, as well as its potential to mitigate the adverse effects associated with this state. For this, we used an in vitro model of IR induced by palmitate (PA), which in other cellular systems has already been described as an inducer of IR [50,51]. To the best of our knowledge, this is the first time an insulin resistance in vitro model has been made using an LC line. The phosphorylation of IRS-1 on Ser^307^ was selected to assess the IR and validate this in vitro model, as previous research with this model indicated disrupted insulin signaling [52]. The chosen phosphorylation of IRS-1 on Ser^307^ occurs naturally after the activation of the insulin receptor in mice (corresponding to the phosphorylation of Ser^312^ in humans), and reduced phosphorylation of this amino acid can lead to the halting of the insulin signaling cascade [52,53]. Hence, p-IRS-1 (Ser^307^) is required for the signaling to occur [52], and our results show that in the presence of PA, there is a reduction in this phosphorylation of IRS-1, even with insulin present (as is the case of our experiments), which may be described as a resistance to the action of this hormone. Concerning this phosphorylation, it has also been reported that hyperinsulinemia may result in the permanent phosphorylation of IRS-1 on Ser^307^, resulting also in the hampering of insulin signaling. This pathway is different from the model we established, which was based on exposure to PA and a decrease in the sensitivity to insulin [52]. In fact, in the initial stages of IR, there seems to be a reduction in serine phosphorylation of IRS-1. Moreover, it has been shown that PA inhibits the insulin cascade by being converted to ceramide, which breaks the pathway by hindering Akt action, which follows IRS-1 in the insulin signaling cascade [54,55]. Our findings show that CrPic_3_ was not capable of halting the impact of PA exposure on p-IRS-1 (Ser^307^) levels in our LC IR model and restoring insulin signaling. While Chen and colleagues suggested that CrPic_3_ could improve IR by decreasing p-IRS-1 (Ser^307^) [56], it has been shown that this decrease is not effectively beneficial to the insulin signaling cascade [52]. Furthermore, under our experimental conditions, CrPic_3_ did not appear to influence insulin signaling through this mediator. In fact, it became evident that cells exposed to PA in conjunction with CrPic_3_ exhibited reduced cell viability compared to those solely exposed to PA. This implies that the use of CrPic_3_ in IR cells may render LCs susceptible to cytotoxic effects.

In addition, we conducted an analysis of the LC exometabolome using ^1^H-NMR spectroscopy to gain insights into how CrPic_3_ affects cellular metabolism and whether it can ameliorate the adverse effects of IR on these cells. Among the metabolites examined, particular attention was given to glucose metabolism. However, LCs from the groups exposed to PA did not show an alteration in the consumption of glucose. The induction of a state of IR did not reduce glucose consumption by LCs, and a possible reason for that could be the fact that in LCs, two glucose transporters (GLUT) have been described—GLUT8 and GLUT9 [57]. While GLUT8 is insulin-responsive, i.e., in a state of IR, it would be downregulated, [58], GLUT9 has been described as insulin-independent, and even though it is more associated with excretion of uric acid [59], it is also associated with the transport of hexoses and can compensate for the lack of other glucose transporters in the absence of insulin or in states of IR [60]. In fact, in our experiments, only the cells exposed to PA and 10 µM CrPic_3_ exhibited altered (lower) glucose consumption when compared to those exposed to only 10 µM CrPic_3_, which indicates that in the presence of higher doses of CrPic_3_, the IR status that was established was able to affect the uptake of glucose.

**Figure 9:**
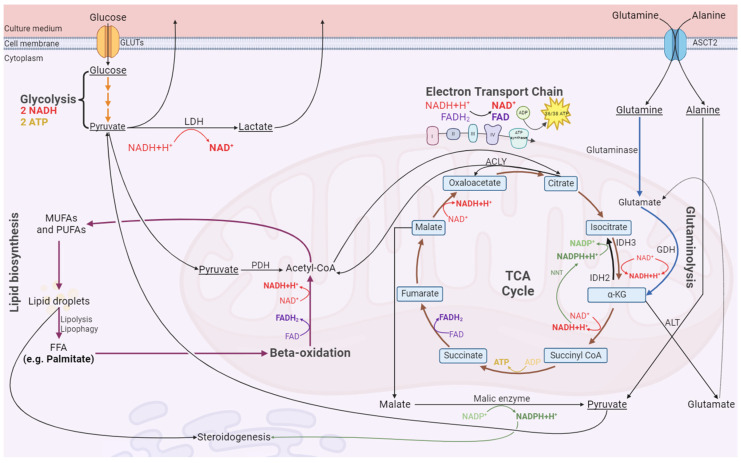
Schematic representation of the proposed mechanisms by which LCs produce pyruvate to be converted into lactate to regenerate NAD^+^. Abbreviations: ACLY—ATP-citrate lyase; ADP—adenosine diphosphate; ALT—alanine aminotransferase; ASCT2—Alanine-Serine-Cysteine Transporter 2; ATP—adenosine triphosphate; FAD^+^—flavin adenine dinucleotide; FADH_2_—reduced Flavin adenine dinucleotide; FFA—free fatty acid; GDH—glutamate dehydrogenase; GLUTs—glucose transporters; IDH—isocitrate dehydrogenase; LDH—lactate dehydrogenase; MUFAs—monounsaturated fat; NAD^+^—nicotinamide adenine dinucleotide; NADH—reduced nicotinamide adenine dinucleotide; NADP^+^—nicotinamide adenine dinucleotide phosphate; NADPH—reduced nicotinamide adenine dinucleotide phosphate; NNT—nicotinamide nucleotide transhydrogenase; PDH—pyruvate dehydrogenase; PUFAs—polyunsaturated fat.

Furthermore, it was intriguing to find that the production of pyruvate, and especially lactate, was increased to levels that exceed the capacity of glycolysis, since one molecule of glucose (C_6_) produces two molecules of pyruvate (C_3_), and each of these can be converted to lactate (C_3_) [44]. Hence, other sources of carbon are being used by these cells to produce the lactate and pyruvate exported, for instance, glutamine and alanine. Table 2 shows that the sources of carbon in non-PA-exposed groups must extend beyond glucose, glutamine, and alanine. We speculate that lactate and the other exported metabolites are generated through glycolysis and glutaminolysis, with the participation of the malic enzyme and alanine aminotransferase (Figure 9). Since there is a marked consumption of glutamine, the hypothesis of glutaminolysis occurring is a strong possibility, considering that various studies revealed that lactate can be generated with glutamine as a source of carbons, with glutamine being metabolized to lactate in human diploid fibroblasts [61], HeLa cancer cells [62], and glioblastoma cells [63]. Intriguingly, Pérez-Escuredo and colleagues reported that lactate stimulates glutaminolysis in cancer cells, as in the case of the LCs established from a testicular tumor [64]. This is a potential mechanism of positive feedback that allows for the generation of energy. Thus, it would be interesting to study if this phenomenon is exclusive to cancer-derived LCs or if it also occurs in LC primary cultures. In glutaminolysis, glutamine is converted by glutaminase into glutamate, which enters the mitochondria, and is converted by glutamate dehydrogenase (GDH) into α-ketoglutarate (α-KG) in an anaplerotic reaction [65]. α-KG can then follow the normal tricarboxylic acid (TCA) cycle or proceed via two different routes: (1) react with alanine and form more glutamate and pyruvate, catalyzed by alanine aminotransferase (ALT) [66], or (2) engage in reactions contrary to the TCA cycle, being converted to isocitrate by isocitrate dehydrogenase (IDH), which in turn is converted to citrate by aconitase, which, when in excess, can be converted into acetyl-CoA and oxaloacetate by ATP–citrate lyase (ACLY) [67]. Concerning oxaloacetate, it can either enter the conventional TCA cycle or, in excess, follow an alternative route of being converted to malate by mitochondrial malate dehydrogenase and then transferred to the cytosol by the dicarboxylate carrier (DIC). In the cytosol, the malic enzyme catalyzes its conversion to pyruvate [68], which can in turn be converted to lactate. The malic enzyme is present in the LC [69], and this reaction generates NADPH (reduced nicotinamide adenine dinucleotide phosphate), which can be used for biosynthesis, namely steroid production [70], making this an important pathway for steroidogenesis to occur in LCs. Another pathway in which NADPH may be involved is the biosynthesis of lipids. In brief, acetyl-CoA is sequentially converted to malonyl-CoA, PA, monounsaturated fatty acids (MUFA), and polyunsaturated fatty acids (PUFA), which are stored in lipid droplets. These reactions are, respectively, catalyzed by acetyl-CoA carboxylase, fatty acid synthase, stearoyl-CoA desaturase, and fatty acid desaturase [71]. Lipid droplets are used for steroid production or to fuel mitochondrial beta-oxidation to originate acetyl-CoA. In opposition, the case of LCs exposed to PA is particularly interesting, given that in these cells, glucose, glutamine, and alanine seem to be enough to provide carbons for lactate and pyruvate, contrary to the cells of the control group or those exposed only to CrPic_3_, which appear to have other carbon sources to produce pyruvate and lactate. This is evident since the carbon ratio of produced/consumed metabolites is close to 1 in cells exposed to PA, as can be seen in Table 2. We hypothesize that PA-exposed cells may metabolize part of PA to regenerate NAD^+^ by desaturating it. This may occur after the conversion of PA to palmitoleic acid by stearoyl CoA desaturase in a pathway similar to that of the pancreatic β-cells [72]. Palmitoleic acid could then be desaturated by delta-5 and delta-6 desaturases (D5D/D6D), with the oxidation of NADH and regeneration of NAD^+^, which would be more available for other reactions, namely glycolysis [73]. In non-PA-exposed cells, this mechanism would not occur, forcing them to resort to the previously described routes to form pyruvate and lactate.

Besides the mentioned uses of glutamine, LCs use it as fuel for steroidogenesis, functioning as a source of energy and carbons in the absence of glucose [74]. No change in glutamine consumption was found, indicating that neither CrPic_3_ nor PA affected the use of glutamine, which may help LCs in cases of IR or Cr(III) intoxication. High glutamine synthase levels in LCs [75] could contradict our results since this would imply an increase in glutamine production, as this enzyme produces glutamine from glutamate. However, this might be a glutamine renewal mechanism, indicating that glutamine consumption might be greater than measured values.

Mitochondria play a pivotal role in LCs, which are the primary sites of testosterone production in the testes. Beyond the generation of energy through oxidative phosphorylation, providing the necessary ATP for steroidogenesis, these organelles are also central to the biosynthesis of steroids, as key enzymes involved in this process are located within the mitochondria [76,77]. Hence, we evaluated the function of the mitochondria by assessing the expression of citrate synthase and mitochondrial electron transport chain complexes (CI-CV), as well as markers for oxidative damage caused by reactive oxygen species (ROS). Citrate synthase is often used as a reliable indicator of mitochondrial content and activity [45]. While PA is known to cause mitochondrial stress in several tissues, like adipose and cardiac tissues, by impairing calcium homeostasis [78], in our experimental conditions, the content and activity of mitochondria of the LCs were not altered by either CrPic_3_ and/or PA, since CS activity remained unchanged in all groups. Regarding the mitochondrial complexes, CII and CIII are associated with ROS production [79,80,81]. Sergi and colleagues revealed that PA upregulated CIII and provoked mitochondrial fragmentation [82]. The results we obtained indicate a noteworthy tendency to increase the expression of CII and CIII in PA-exposed LCs, while the combination of PA and CrPic_3_, especially in higher concentrations, restores the expression of CII and CIII to control levels. The effects of CrPic_3_ on these complexes have never been studied, whereas, as mentioned, 500 μM of PA has been described to increase CIII and cause the aforementioned mitochondrial fragmentation [82]. Interestingly, these findings align with the results we obtained when evaluating lipid peroxidation in these cells, given that PA significantly increased lipid peroxidation and CrPic_3_ normalized this oxidative stress hallmark to control levels. As discussed in previous sections, CrPic_3_ has been shown to decrease lipid peroxidation [24,25,27], an effect we also observed in this study. In turn, contradictory information was found regarding the effect of PA. Palomino and colleagues described that the same concentration used in this study decreased lipid peroxidation in vascular endothelial cells [83], whilst Haffar and colleagues reported that 500 μM of PA did not affect lipid peroxidation but increased ROS production [84]. Our results indicate that, in murine LCs, PA tends to increase the expression of CII and CIII and most likely causes some of the effects already described, such as an increase in ROS [85]. Another hypothesis we propose is that the presence of PA and CrPic_3_ may be overburdening the mitochondria. Koves and colleagues showed that exposure to 500 μM of FFA such as PA increases beta-oxidation in the mitochondria and that in the absence of physical exercise, the TCA cycle is hindered by redox pressure and intermediate depletion, which leads to incomplete beta-oxidation and accumulation of toxic metabolites [86]. Hence, the decrease seen in the abundance of CII and CIII in CrPic_3_ and PA-exposed groups may be due to toxic effects. Even though the concentration of PA is lower than the one used in this study, the combination of CrPic_3_ may potentiate the same effect, given that our findings showed that this combination hinders metabolic viability in LCs. Cr(III) is known to inhibit CV and increase the AMP/ATP ratio, potentially reducing mitochondrial function during stress [87]. Our results show that a combination of PA and 10 μM of CrPic_3_ inhibits the expression of this complex. This suggests that the previously observed inhibition extends beyond functionality to affect complex expression.

Regarding steroidogenesis, it would be expected that CrPic_3_ could have an impact on it, since Navin and colleagues showed that Cr(III) reduces crucial enzymes for steroid biosynthesis [88]. However, our results show that CrPic_3_ by itself does not improve or hinder androstenedione production in the chosen LC. In fact, the results show that in the PA-exposed cells, 10 μM of CrPic_3_ increases steroidogenesis. A possible explanation for this is that IR might be associated with increased cholesterol metabolism, as was described in liver cells [89], with the same occurring in LCs, as they are capable of carrying out cholesterol biosynthesis [90]. A meta-analysis discussed the fact that CrPic_3_ is important for the lowering of cholesterol levels in the blood, showing that it may play a role in regulating the cellular lipid profile [91]. With the accumulation of cholesterol, CrPic_3_ may induce its utilization in LC steroidogenesis by mechanisms not yet characterized.

Overall, our results demonstrate that high concentrations of CrPic_3_ (100 µM) are toxic to LCs, and the continued administration of doses that lead to an accumulation of these levels in the testes is inadvisable. At lower concentrations, CrPic_3_ does not damage the metabolic viability of LCs, although, in the IR in vitro model, it prompted a cytotoxic effect. Even though it is sold for its antidiabetic properties, CrPic_3_ failed to improve glucose consumption in LC-affected IR or even under control conditions. Yet, it did reduce oxidative stress in cells subjected to IR, although it was not clear if this effect came from toxic effects, as it reduced metabolic viability in this condition. Additionally, CrPic_3_ did not affect basal androgen production but, when in the presence of PA, prompted an increase in the production of androstenedione, which may be linked to a promotion of cholesterol utilization in LCs. Regardless, it is important to consider that we used the BLTK1 cell line, an LC line immortalized from testicular cancer. Even though this type of model is critical for expanding our understanding of this subject, it is worth mentioning that these cells are different from primary cultures or in vivo cells. One notable difference is the ability to perform cell division, which does not occur in adult LCs. Hence, the metabolism of these cells may diverge from the aforementioned cells, which stands as the main limitation of the present study. Nevertheless, we maintain that despite this limitation, our study provides valuable insights into the effects observed in LCs exposed to CrPic_3_, including in cases of insulin resistance. Another limitation in our study stands from the fact that the metalloestrogen effect of Cr(III) was not considered, even though this could lead to alterations in the modulation of endocrine pathways involving estrogens, which are tightly related to androgens [92]. Future studies should elucidate this topic.

## 5. Conclusions

CrPic_3_ is used as a nutritional supplement for weight loss, glucose control, and oxidative stress reduction. However, safety considerations, particularly regarding male reproductive health, have been raised. Some concerns surround its impact on androgen-producing LCs, so to study the repercussions of long-term exposure to CrPic_3,_ we incubated the LC line BLTK1 to mimic the exposure to low and high accumulations of this compound in the testes. Moreover, a model where BLTK1 became resistant to the action of insulin was established using PA to study the antidiabetic properties of that compound in these cells.

Cytotoxicity assays showed that after 24 h of exposure to high concentrations of CrPic_3_, it prompted cytotoxic effects in LCs, and while PA alone showed no cytotoxicity, CrPic_3_ and PA combined also caused a decrease in LC metabolic viability. Furthermore, CrPic_3_ was not able to restore the insulin insensitivity status prompted by PA or increase glucose consumption, not exhibiting the proclaimed antidiabetic effect in these cells. Still, it presented an antioxidant effect by decreasing lipid peroxidation. Finally, unlike what was reported in other studies, CrPic_3_ alone did not change steroid production, but when in the presence of PA, it served as an inducer of steroid production.

Future work should include more studies on LC lines, namely on other effects on mitochondria and on antioxidant enzymes, and clarify some unknown mechanisms, such as those resulting in the production of lactate, the relevance of each GLUT in the uptake of glucose, and why CrPic_3_ only increases steroidogenesis in the presence of PA when PA does not change steroid production by itself. Moreover, the long-term effects of CrPic_3_ on LCs could be investigated. Other studies could expand to other cell types related to male fertility, such as Sertoli, sperm, and germ cells. If the results warranted it, conducting in vivo studies, pharmacokinetic research, and clinical trials could become an option for further research.

In summary, this work demonstrates that at high concentrations, CrPic_3_ poses a danger for LCs, and its antidiabetic properties are not observed in these cells. Even though some antioxidant effects are evident, it remains unclear if these are a consequence of unstudied toxicity. Further work is still needed to determine if CrPic_3_ is safe for the reproductive health of men. This study is a clear stride forward toward unveiling its effects, yet exercising caution is recommended when contemplating the use of CrPic_3_ as a nutritional supplement.

## Figures and Tables

**Table 1:** List of primary and secondary antibodies used for Western and slot-blot.

Target	Catalog Number	Host Species	Predicted Molecular Weight (kDa)	Dilution	Vendor
p-IRS-1 (Ser307)	#2381	Rabbit	132	1:500	Cell Signaling, Massachusetts, USA
OXPHOS	ab110413	Mouse	20, 30, 40, 48, and 55	1:2000	Abcam, United Kingdom
4-HNE	AB5605	Goat	n.a.	1:1000	EMD Millipore, California, USA
DNP	D9656	Rabbit	n.a.	1:5000	Sigma-Aldrich, St. Louis, Missouri, USA

Abbreviations: 4-HNE—4-hydroxynonenal; DNP—2,4-dinitrophenyl; p-IRS-1—phosphorylated insulin receptor substrate 1; n.a.—not applicable; OXPHOS—oxidative phosphorylation; Ser^307^—serine 307.

**Table 2:** Ratio of carbon equivalents (Cs) between produced and consumed metabolites to understand the contribution of glucose, glutamine, and alanine to the production of lactate and pyruvate.

(CrPic3) (µM)	(PA) (µM)	Metabolites Consumed			Sum of Cs Consumed *	Metabolites Produced		Sum of Cs Produced	Ratio of Cs Produced vs. Consumed
		Glucose	Glutamine	Alanine		Lactate	Pyruvate		
0	0	21.59	160.2	13.41	650.37	407.4	34.12	1324.56	2.04
0.1	0	20.04	184.5	7.53	696.33	384.4	35.11	1258.53	1.81
10	0	27.46	187.8	11.25	761.91	303.1	28.93	996.09	1.31
0	250	24.93	198.9	10.29	777.15	221.7	43.3	795.00	1.03
0.1	250	31.29	231.7	13.12	922.20	249.5	43.21	878.13	0.95
10	250	15.05	178.1	16.77	674.91	234.8	18.36	759.48	1.13

Legend: * the carbon equivalents of glutamine were adjusted to 3 when considering its conversion to pyruvate.

## Data Availability

Data are contained within the article.
